# Resonant Raman spectroscopy elucidates pH-reversible chelation of blueberry anthocyanins with metal ions and carrageenan-enhanced photothermal stability

**DOI:** 10.1016/j.fochx.2026.103781

**Published:** 2026-03-22

**Authors:** Yixiao Wang, Menglong Ma, Haitao Fu, Xianhao Ding, Chuchu Duan, Xiaodan Liu, Huang Dai, Ning Yang, Fuwei Pi, Jiahua Wang, Ming Zhang

**Affiliations:** a School of Food Science and Engineering, Wuhan Polytechnic University, Wuhan 430023, Hubei, China; b School of Food and Health, Beijing Technology & Business University, Beijing 100048, China; c School of Food Science and Technology, Jiangnan University, Wuxi 214122, Jiangsu, China

**Keywords:** Blueberry anthocyanins, Metal chelation, Carrageenan stabilization, Raman spectroscopy, Degradation kinetics, pH-dependent reversibility

## Abstract

Blueberry anthocyanins (BA) are commonly incorporated into foods and beverages to enhance their functionality, and the stability of BA in metal-ion-rich systems has attracted considerable attention. The structural dynamics and stability of BA-metal ion complexes were investigated using resonant Raman spectroscopy combined with Gaussian multi-peak fitting techniques. BA form pH-reversible chelates with Fe^2+^ or Zn^2+^, characterized by distinct Raman peaks at 1524 cm^−1^ and 1640 cm^−1^, respectively. Adding carrageenan (CG) significantly improved the complexes' stability. During thermal treatment at 80 °C, the degradation of CG-stabilized complexes followed second-order kinetics, with rate constants (0.0001–0.0002 min^−1^) only one-fifth to one-tenth of the unstabilized system (0.001 min^−1^). Under UV light, degradation was zero-order, with rates (0.0014–0.0039 h^−1^) also significantly lower than the control (0.0097 h^−1^). In conclusion, this study demonstrates that CG-stabilized anthocyanin-metal complexes exhibit enhanced stability, offering promising applications in functional foods, with Raman spectroscopy serving as an effective tool for real-time monitoring.

## Highlights

• pH-reversible chelation of blueberry anthocyanins with Fe^2+^ and Zn^2+^.
• Characteristic Raman peaks at 1524 cm^−1^ and 1640 cm^−1^ identify Fe^2+^/Zn^2+^ chelation.
• Carrageenan greatly enhances thermal and UV stability of anthocyanin-metal complexes.
• Carrageenan slows heat (2nd-order) and UV (zero-order) degradation of chelation.
• Resonance Raman combined Gaussian fitting monitors anthocyanin structural dynamics.

## 1 Introduction

Anthocyanins are a class of water-soluble natural pigments widely found in plants and belong to the flavonoid family (Mozos et al., 2021). These compounds impart rich colors—ranging from red and purple to blue—to fruits, vegetables, and flowers (Kan et al., 2022). They are commonly present in foods such as blueberries, grapes, red cabbage, blackberries, purple sweet potatoes, and cherries (Lianza et al., 2022). Anthocyanins are recognized as natural bioactive ingredients with remarkable health-promoting properties. Their multiple benefits—including antioxidant, anti-inflammatory, cardioprotective, and neuroprotective effects—have been widely supported by scientific research (Avula et al., 2023). Incorporating anthocyanin-rich foods into one's daily diet is an effective strategy for promoting long-term health.

Blueberries are among the botanical sources with the highest content of the antioxidant anthocyanin. Blueberry anthocyanins (BA) can be added to food and beverages to create new functional items (Wu, Han, et al., 2023). Beneficial trace metal ions such as Fe^2+^/Fe^3+^ and Zn^2+^ are widely present in food systems and can form chelates with anthocyanins. The color of anthocyanins depends greatly on their structure and the pH of the environment (Abedi-Firoozjah et al., 2022). After binding with metal ions, the solution color often shifts from red to blue-purple (Mollaamin et al., 2021), and in some cases may even form precipitates, reducing the sensory appeal of the product. More importantly, chelation can alter the degradation pathway and rate of anthocyanins. Through rational design, the metal–anthocyanin interaction can be utilized to enhance or improve product functionality (Weiss et al., 2024; Wu, Fan, et al., 2023). This provides a scientific basis for developing mineral-fortified beverages or foods rich in anthocyanins.

However, anthocyanins are sensitive to light, heat and pH, exhibiting greater stability under acidic conditions (Cai et al., 2022; Herrera-Balandrano et al., 2021; Uzun et al., 2025). They are also prone to degradation during processing and storage. To address challenges such as color instability resulting from chelation, modern food technology often employs strategies like microencapsulation or the use of copigments (Rosales et al., 2023). Among these, polysaccharides (such as gum arabic and modified starch) and organic acids (such as citric acid and ascorbic acid) have proven particularly effective. These compounds not only compete with metal ions for binding, thereby stabilizing the natural color of anthocyanins, but also physically encapsulate and protect anthocyanin molecules through the formation of more complex three-dimensional network structures, thereby maximizing the retention of their health benefits (Yan et al., 2025). An inulin-based microencapsulation technique was employed to enhance the photothermal stability of strawberry anthocyanins (Gomes et al., 2021). In a related study, ferulic acid and caffeic acid were introduced into anthocyanin-rich mulberry extracts, which resulted in increased stability and color intensity of the anthocyanins, thereby improving the functionality of the processed products (Chen et al., 2022). However, research on the photothermal stability and degradation kinetics of anthocyanin-metal ion complexes enhanced by such additives remains insufficient.

Current research on the stability and degradation kinetics of anthocyanins and their metal complexes (BA–M^n+^) primarily relies on high-performance liquid chromatography (HPLC) and spectrophotometry (Tang & Giusti, 2020). HPLC requires complex sample pretreatment, which may lead to loss or degradation of anthocyanins, while spectrophotometry can only provide absorbance information and cannot accurately analyze changes in chemical bonds or functional groups. As fingerprint spectroscopic techniques, infrared (IR) and Raman spectroscopy are widely used in food analysis (Jeyaram & Geethakrishnan, 2020). However, due to strong water absorption in the IR region, which masks other characteristic peaks (Stuppner et al., 2020), IR spectroscopy is not suitable for aqueous systems. In contrast, water does not exhibit a characteristic Raman shift, which enables Raman spectroscopy to yield detailed chemical and structural molecular information (Carvalho et al., 2019). Therefore, Raman spectroscopy has been applied for the quantitative analysis and photothermal stability assessment of anthocyanins in aqueous systems (Duan et al., 2024). Moreover, the use of 532 nm excitation wavelength induces a strong resonance Raman effect, as it falls within the absorption band of anthocyanins. Resonance Raman spectroscopy has also been used to detect lycopene, since the same excitation wavelength (532 nm) lies within the absorption range of lycopene (300–550 nm) (Hara et al., 2018). Similarly, utilizing the resonance or pre-resonance Raman effects can enhance the intensity of anthocyanin Raman peaks, which is highly beneficial for analyzing anthocyanin degradation in complex systems. However, no studies have yet reported the use of confocal Raman spectroscopy to investigate the stability and degradation kinetics of BA-metal ion chelates.

The development of mineral-fortified beverages and foods rich in anthocyanin represents a significant application of blueberry anthocyanin extract (BAE). In this study, BA-Fe^2+^/Zn^2+^ chelates were prepared and stabilized using carrageenan (CG). The chelate systems were subjected to *v*arious heat treatment times and ultraviolet (UV) irradiation durations. By combining resonance Raman spectroscopy with Gaussian multi-peak fitting, the formation of pH-reversible chelates between BA and Fe^2+^ or Zn^2+^ was demonstrated, and the photothermal stability and degradation kinetics of the BA–metal ion complexes were quantified.

## 2 Materials and methods

### 2.1 BA solution preparation

The commercially available BAE used in this study was obtained through water-ethanol extraction and contained 25% anthocyanins. The BAE was dissolved in ultrapure water and stirred with a magnetic stirrer at 600 rpm for 20 min to prepare a 0.25% (*w*/*v*) BA aqueous solution (BAAS). This solution was stored at 4 °C in the dark. The initial pH of the BAAS was measured as 2.93 using a Mettler-Toledo standard pH meter.

#### 2.1.1 BA-Fe^2+^(Zn^2+^) chelation

A 20 mL aliquot of the prepared BAAS was accurately measured and transferred into a beaker. Then, 0.01 g, 0.02 g, 0.03 g, 0.04 g, and 0.05 g of food-grade ferrous gluconate (C_12_H_22_FeO_14_, 98.20% on dry basis) (FG) and zinc gluconate (C_12_H_22_O_14_Zn, 99.57% on dry basis) (ZG) solids were added respectively. The mixture was stirred at room temperature using a magnetic stirrer set at 600 rpm for 20 min to ensure complete dissolution, producing aqueous solutions of BA-Fe^2+^ and BA-Zn^2+^ chelates.

#### 2.1.2 pH adjustment of aqueous solutions of BA-Fe^2+^(Zn^2+^) chelates

The pH of the BA-metal ion chelate aqueous solutions was adjusted using analytical-grade sodium bicarbonate and citric acid. A 20 mL aliquot of the metal ion-BA chelate solution was transferred to individual beakers. Sodium bicarbonate or citric acid was then gradually added to adjust each solutions to its target pH. The pH was continuously monitored during adjustment using a Mettler-Toledo standard pH meter.

### 2.2 External perturbation of BAAS+FG(ZG) + CG

#### 2.2.1 Preparation of BAAS+FG(ZG) + CG

To prepare the BA-metal ion chelate solution, 0.05 g of FG or ZG was added to BAAS, to which 0.06 g of CG was then added. The mixture was homogenized at 60 °C with constant magnetic stirring at 600 rpm for 20 min to ensure complete hydration of CG.

#### 2.2.2 Temperature perturbation

A temperature of 80 °C was selected to simulate the thermal conditions common in food processing, such as pasteurization or hot filling, enabling efficient evaluation of thermal stability. Previous studies have confirmed that anthocyanins undergo significant thermal degradation above 65 °C, particularly beyond 80 °C (Duan et al., 2024). Four 5 mL aliquots of the ternary complexes were heated in an 80 °C constant-temperature digital water bath (1200 W, Model WB100–6, Changzhou Osai Instrument Factory, China). Samples were collected after 1, 2, 3, and 4 h of heating, respectively. After cooling to ambient temperature (24 °C), Raman spectra were acquired.

#### 2.2.3 Ultraviolet irradiation

The ternary complex solutions were aliquoted into 5 mL glass vials and placed in light-tight containers. Continuous irradiation was performed using a 10 W UV-LED lamp (emission spectrum: 380–420 nm, peak wavelength: 395 nm; Shenzhen Zhonglian Guangcai Factory, China). Samples were collected at 24-h intervals over a total irradiation period of 144 h for Raman spectral analysis, resulting in six sampling timepoints.

#### 2.2.4 Zeta potential measurement

The zeta potential of the samples was measured using a Zeta Potential Analyzer (Model BeNano 180 Zeta Pro, Bettersize Instruments Ltd., China). Each sample was diluted fivefold with deionized water prior to measurement. Three replicate measurements were performed for each sample, and the results are reported as the mean values.

### 2.3 Resonance Raman spectroscopy measurement

Raman spectra were acquired using a confocal micro-Raman spectrometer (Model inVia Qontor, Renishaw Co., London, UK). The system was equipped with a Leica microscope system featuring 10× eyepieces and a 50× long working distance objective, a 1024 × 256 pixel charge-coupled device detector, selectable gratings (1800 or 1200 lines/mm), and laser sources at 532 nm (rated power approximately 50 mW), 633 nm (approximately 17 mW), and 785 nm (approximately 300 mW). Instrument control was performed using WiRE 5.3 software (Renishaw Co., London, UK).

Anthocyanins exhibit UV–Visible absorption between 280 and 550 nm. Therefore, 532 nm excitation produced BA Raman spectra with a higher signal-to-noise ratio and more pronounced spectral intensity compared to 633 or 785 nm excitation, facilitating the collection of resonance or pre-resonance Raman spectra from the solution system. Based on this, the 532 nm laser was selected with the following parameters: laser power set to 5% of maximum (approximately 2.5 mW), single exposure time of 10 s, three accumulated scans, and a spectral range of 300–1800 cm^−1^.

### 2.4 Chemometrics and statistical analysis

#### 2.4.1 Spectral pre-processing

Raman spectral preprocessing was performed using WiRE 5.3 software (Renishaw Co., London, UK). To improve the signal-to-noise ratio, a sequential preprocessing protocol was applied (Duan et al., 2024). First, spike correction was used to remove random spike artifacts caused by cosmic rays or detector noise. This procedure identifies and eliminates isolated wavenumber points exhibiting intensity deviations significantly beyond adjacent data points while remaining below a set threshold. Next, baseline correction was executed using polynomial fitting. Finally, noise reduction was achieved with a Savitzky-Golay smoothing filter while preserving key spectral peak features.

#### 2.4.2 Gaussian multi-peak fitting

Gaussian multi-peak fitting essentially deconvolves chemical information from instrumentally overlapping signals, providing molecular-level structural resolution in complex systems (Wang et al., 2021). In this study, the technique was indispensable for revealing structure-activity relationships in the ternary complexes, especially for quantifying the effects of microscopic interactions. Peak fitting was performed using Gaussian functions in OriginPro software (OriginLab, Northampton, MA, USA) for stoichiometric analysis and scientific graphing. The goodness of fit for the Gaussian deconvolution was assessed using the coefficient of determination (R^2^).

#### 2.4.3 ANOVA and Pearson correlation analysis

One-way analysis of variance (ANOVA) was performed using Origin Pro 2021 (OriginLab, Northampton, MA, USA). The data are expressed as mean ± standard deviation (SD). Statistical significance was defined as follows: *P* > 0.05, not significant; 0.01 < *P* ≤ 0.05, significant; and *P* ≤ 0.01, highly significant.

The Pearson correlation coefficient, which assesses the relationship between two variables based on their covariance matrix, is commonly used to explore overall variable associations. In this study, it was applied to examine relationships between Raman feature variables and their correlations with FG/ZG dosage. Correlation direction (positive/negative) and strength (magnitude of *R*) were visualized using a correlation heatmap. Variables showing the highest correlations were selected for quantitative analysis. All correlation analyses were performed in the R software environment.

### 2.5 Degradation kinetics

Degradation kinetics were employed to investigate the rate and pattern of the reduction in BA concentration over time. Zero-order, first-order, and second-order kinetic models were evaluated, and the best-fitting model was selected by comparing the R^2^ for each. Linear regression was applied to most suitable model to determine the rate constant (*k*) from its slope and intercept. Based on the *k* values, the protective effect of CG against the degradation of BA-Fe^2+^/Zn^2+^ chelates under thermal treatment and UV irradiation was evaluated and compared.

### 2.6 Degradation rate

The characteristic Raman peak at 1567 cm^−1^, corresponding to the C—C tensile vibration of the benzopyran ring in anthocyanin-based compounds, was selected and normalized against the peak intensity at 631 cm^−1^ (Duan et al., 2024), which is associated with the stable in-plane bending vibration of the C—C single bond. The resulting peak intensity ratio obtained from multi-peak fitting was used to evaluate changes in the chelate structure under heat treatment or UV irradiation. Furthermore, the degradation rate was applied to compare the degradation behavior of CG-stabilized BA-Fe^2+^/Zn^2+^ chelates under these perturbations. The degradation rate was calculated using the following formula:

(1) $$\text{Degradation rate}=\frac{Y_{n+1}-Y_{n}}{Y_{n}}\times 100\%$$

where *Y*_n_ is the peak intensity ratio (*I*_1567_/*I*_631_) of the chelate at the *n*th processing sequence and *Y*_*n+*1_ is the peak intensity ratio at the (*n* + 1)th processing sequence.

## 3 Results and discussion

### 3.1 Raman characterization of BA-Fe^2+^(Zn^2+^) chelates

**Fig. 1:**
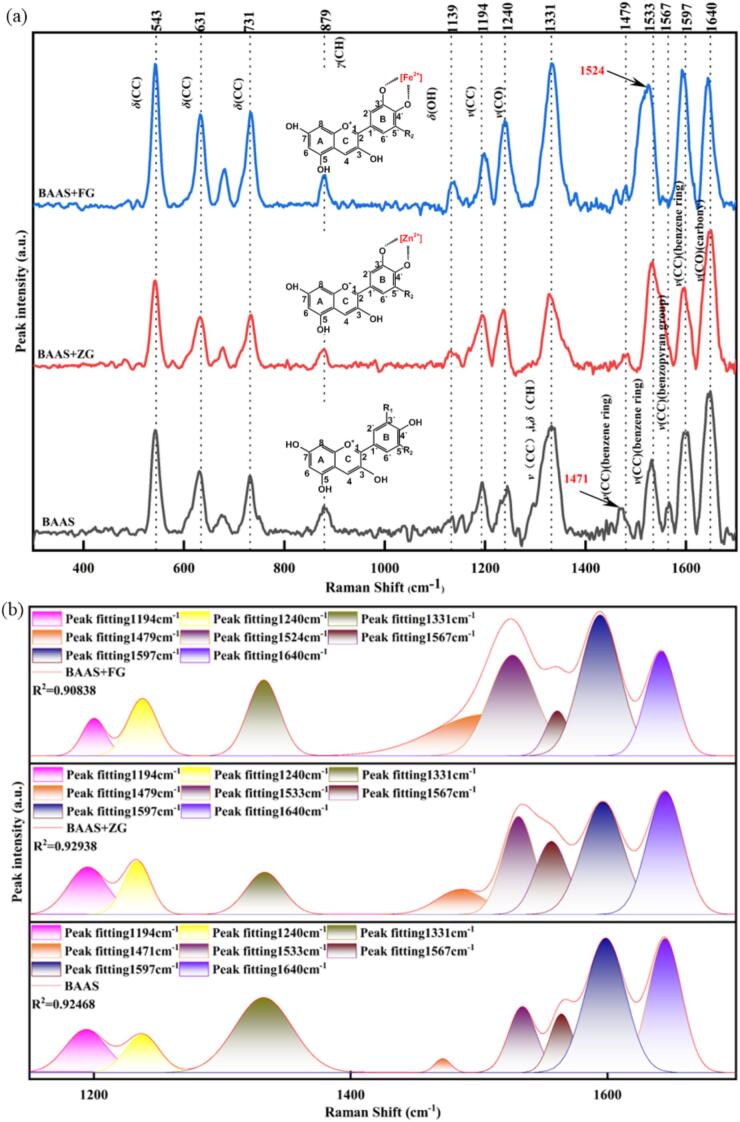
Raman spectra of blueberry BAAS, BAAS+FG and BAAS+ZG (a) and Gaussian multi-peak fitted spectra in the range of 1100–1700 cm^−1^ (b).

Chelation between anthocyanins and metal ions involves intermolecular charge transfer, which metal ions accept electron donors from the phenolic hydroxyl groups of anthocyanins through their vacant orbitals, forming stable chelates and resulting in a bathochromic shift in the spectrum (Zhao et al., 2025). A key structural requirement for effective chelation is the presence of adjacent free phenolic hydroxyl groups (—OH) on the B ring, a feature exemplified in the molecular structures of cyanidin (CYN, R_2_: H), delphinidin (DEL, R_2_: OH), and petunidin (PET, R_2_: OCH₃) (Fig. 1(a). In this study, using FG and ZG as model systems, the characteristic peaks of BA-Fe^2+^/Zn^2+^ chelates were characterized and resolved by Raman spectroscopy combined with Gaussian multi-peak fitting.

Chelates were prepared by mixing 0.05 g of either FG or ZG with 20 mL of BAAS. The resulting systems had pH values of 3.74 and 4.02, respectively. The Raman spectra of BAAS, BAAS+FG, and BAAS+ZG are shown in Fig. 1(a), and the characteristic Raman shifts, along with their corresponding functional groups and vibration modes, are detailed in Fig. 1. The characteristic peaks of BAAS largely overlap with those of the chelates, indicating that the primary structure of anthocyanin remains unchanged during chelation. However, in the BAAS+FG system, a significantly enhanced characteristic peak appears at 1524 cm^−1^, which is assigned to the in-plane deformation vibration of the benzene ring (Duan et al., 2024) and a complex coupling involving inter-ring C=C stretching and ring vibration modes. Therefore, the Raman shift at 1524 cm^−1^ can be considered as a characteristic peak for investigating the chelation mechanism of BA-Fe^2+^. This enhancement mechanism arises from the chelation between Fe^2+^ and the ortho-dihydroxyl groups on the B ring of anthocyanin. Concurrently, the solution color changed from purple-red to dark blue, which is attributed to the competition of multivalent metal ions (M^n+^) for protons, driving the transformation of the flavylium cation (red form) to the quinoidal base (blue form) (Tindal et al., 2024). Ultimately, a blue-black precipitate formed, suggesting that Fe^2+^ not only alters the electronic configuration through coordination but may also induce anthocyanin degradation and color instability.

In contrast, the BAAS+ZG system exhibited a distinct response. Although the overall Raman spectral profile did not change significantly (Fig. 1(a)), the relative intensity of the carbonyl (C=O) stretching vibration (*ν* (C=O)) at 1640 cm^−1^ was noticeably enhanced. This supports the potential of the Raman shift at 1640 cm^−1^ to serve as a characteristic variable for monitoring the Zn^2+^-BA chelation process. Furthermore, the characteristic peak at 1471 cm^−1^, attributed to the benzene ring stretching vibration, shifted to 1479 cm^−1^ due to metal ion chelation. This shift is consistent with the behavior observed in the BAAS+FG system, though the effect was more pronounced with Fe^2+^ due to its higher reactivity. Notably, no color change or precipitate formation was observed in the BAAS+ZG system, which is consistent with findings reported by Luna-Vital et al. (2018).

The characteristic Raman shifts were used as fitting variables for Gaussian multi-peak fitting. Within the range of 1100–1700 cm^−1^, the characteristic single peaks and their intensity variations were more pronounced (Fig. 1(b)), and the correlation coefficients (R^2^) between the fitted spectra and the original spectra ranged from 0.91 to 0.93. Gaussian multi-peak fitting was performed to focus on the main characteristic peaks, thereby eliminating the effects of peak overlap and facilitating subsequent quantitative analysis.

### 3.2 Chelation mechanism of BA-Fe^2+^(Zn^2+^) based on Raman spectroscopy

**Fig. 2:**
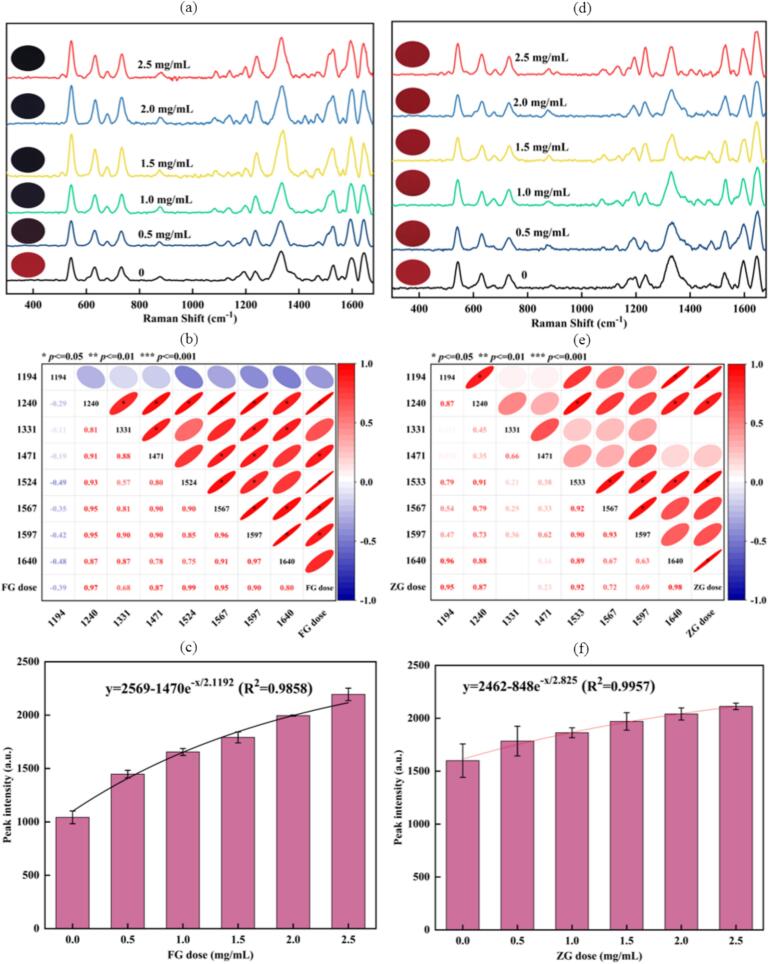
Raman spectra of BAAS+FG (a) and BAAS+ZG (d) at different dosages, Pearson correlation coefficients between FG (b) and ZG (e) dosages, and characteristic Raman peak intensities at 1524 cm^−1^ and 1640 cm^−1^ along with their fitted dependence on FG (c) and ZG (f) dosages, respectively.

Fig. 2(a) illustrates the Raman spectral changes of the BA-Fe^2+^ chelate formed at varying FG concentrations. The peak intensity at 1524 cm^−1^ increased significantly with higher FG dosage, unequivocally identifying this band as the characteristic vibrational mode of the Fe^2+^-BA chelate. Upon the initial addition of FG, the solution color shifted from the original purple-red to purple. As the Fe^2+^ concentration continued to rise, the solution turned blue and eventually transitioned to a blue-black hue (see Fig. 2(a)). The sequential color evolution—from purple to blue to blue-black—confirms the gradual conversion of the flavylium cation to the quinonoidal base (Tindal et al., 2024). Pearson correlation analysis between all characteristic peaks and FG concentration (Fig. 2(b)) revealed that the correlations for peaks at 1240, 1524, 1567, and 1597 cm^−1^ all exceeded 0.9. The peak at 1524 cm^−1^ exhibited the highest Pearson correlation coefficient (0.99) with FG dosage. The quantitative relationship between the peak intensity at 1524 cm^−1^ and the FG addition was described by the equation y=2569−1470e−x2.1192(R^2^ = 0.9858) (Fig. 2(c)), indicating an exponential saturation trend in chelate formation.

Fig. 2(d) displays the Raman spectra of Zn^2+^-BA chelates formed at various ZG concentrations, indicating minimal spectral changes. All aqueous solutions of Zn^2+^-BA chelates exhibited a purple-red color, with no visually distinguishable differences among the varying ZG doses. Pearson correlation analysis between the characteristic peak intensities and ZG concentrations (Fig. 2(e)) revealed correlation coefficients above 0.9 for peaks at 1194, 1533 and 1640 cm^−1^. The peak at 1640 cm^−1^, corresponding to the *ν* (C=O) vibration, exhibited the highest Pearson correlation coefficient (0.98) with ZG dosage, establishing it as a distinctive marker for Zn^2+^-BA chelation. The quantitative relationship between the peak intensity at 1640 cm^−1^ (*y*) and the ZG dosage (*x*) followed an exponential saturation model, y=2462−848e−x2.825 with an R^2^ value of 0.9957 (Fig. 2(f)).

The analysis of the exponential saturation model parameters revealed that the interaction between BAAS+FG exhibits faster binding kinetics, a broader interaction profile, and more pronounced structural changes than that of BAAS+ZG. These differences can be mainly ascribed to the distinctive ionic properties of Fe^2+^ and Zn^2+^, including factors such as ionic radius, charge density, and ligand field strength. In particular, the inherent redox activity of Fe^2+^ likely enables stronger and more complex redox-mediated coordination with reducing anthocyanin molecules. As a result, the Fe^2+^–anthocyanin system displays improved binding characteristics in both thermodynamic and kinetic aspects. Under low-concentration conditions, the chelation efficiency exhibited a positive correlation with metal ion concentration, aligning with the Law of Mass Action. In summary, the quantitative Raman responses at 1524 cm^−1^ (Fe^2+^) and 1640 cm^−1^ (Zn^2+^) effectively provided molecular-level insights into the coordination mechanisms of anthocyanin–metal interactions.

### 3.3 pH-reversible chelation of BA-Fe^2+^(Zn^2+^)

#### 3.3.1 pH-dependent chelation reversibility

**Fig. 3:**
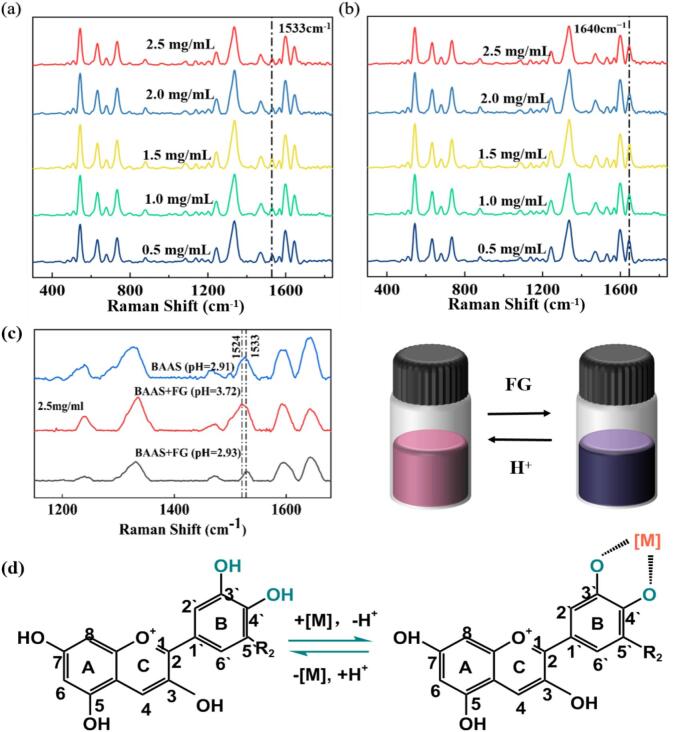
Raman spectra of BAAS+FG (a) and BAAS+ZG (b) at pH = 2.5 with varying additive concentrations, Raman spectroscopy and color development of BAAS+FG under pH adjustment (c), and reversible reaction equation for anthocyanin-metal chelation (d).

The reversibility of BA chelation with Fe^2+^ and Zn^2+^, which is central to their potential application, was clearly demonstrated through systematic pH adjustment. Initial chelation of BAAS with varying doses of FG or ZG resulted in a dose-dependent increase in system pH. Subsequent acidification of these systems to approximately pH = 2 using citric acid effectively reversed the chelation process. Raman spectroscopic analysis confirmed this reversal: in the BAAS+FG system, the characteristic Fe^2+^-chelation peak at 1524 cm^−1^ disappeared and was replaced by the original BAAS peak at 1533 cm^−1^ across all FG concentrations tested (Fig. 3(a)); similarly, in the BAAS+ZG system, the enhanced intensity of the *ν* (C=O) peak at 1640 cm^−1^, indicative of Zn^2+^ interaction, decreased to the baseline level observed in pure BAAS (Fig. 3(b)).

The reversible cycle was detailed using the more visually responsive BAAS+FG system as a representative model (Fig. 3(c)). The addition of 2.5 mg/mL FG to the red BAAS solution (pH = 2.91) induced the formation of a BA–Fe^2+^ chelate, turning the solution blue-black (pH = 3.72) and giving rise to the characteristic Raman peak at 1524 cm^−1^. Subsequent acidification of the mixture with citric acid reversed the color from blue-black back to the original red, accompanied by a Raman peak shift from 1524 cm^−1^ back to 1533 cm^−1^. To further confirm the reversibility, re-alkalization of the system by adding sodium bicarbonate caused the solution color to change again from red back to blue-black, re-establishing the 1524 cm^−1^ peak. The underlying mechanism involves the competition between H^+^ and Fe^2+^ for the ortho-dihydroxy binding sites on the anthocyanin B-ring. Under weakly acidic conditions, Fe^2+^ chelates with the deprotonated phenolic groups, forming the quinonoidal base structure. The introduction of a high concentration of H^+^ from citric acid displaces Fe^2+^, reforming the stable red flavylium cation. Conversely, raising the pH reduces the H^+^ concentration, allowing the phenolic hydroxyl groups on the anthocyanin molecules to deprotonate again. The resulting phenolate anions then re-coordinate with the Fe^2+^ ions in solution, reforming the stable five- or six-membered ring chelate. This reversible binding, modulated by environmental pH and illustrated by the reaction equation in Fig. 3(d), controls the interconversion between the flavylium cation and the quinoidal base.

In summary, BA, as a natural pigment, exhibits color properties that are influenced not only by pH but also significantly modulated by metal ions (metallochromism). This provides a fundamental basis for its potential applications in intelligent packaging and chemical sensing.

#### 3.3.2 Spectral characterization of chelates across a broad pH range

To characterize the spectral properties of the chelates over an extensive pH range, a systematic investigation was conducted. The BAAS+FG and BAAS+ZG systems (2.5 mg/mL), with initial pH values of 3.72 and 4.01, respectively, were adjusted to pH levels ranging from 2.0 to 8.0 in increments of 1 pH unit (ΔpH ±0.15). The corresponding Raman spectra (1100–1700 cm^−1^) were then deconvoluted using Gaussian multi-peak fitting and further analyzed by principal component analysis (PCA).

**Fig. 4:**
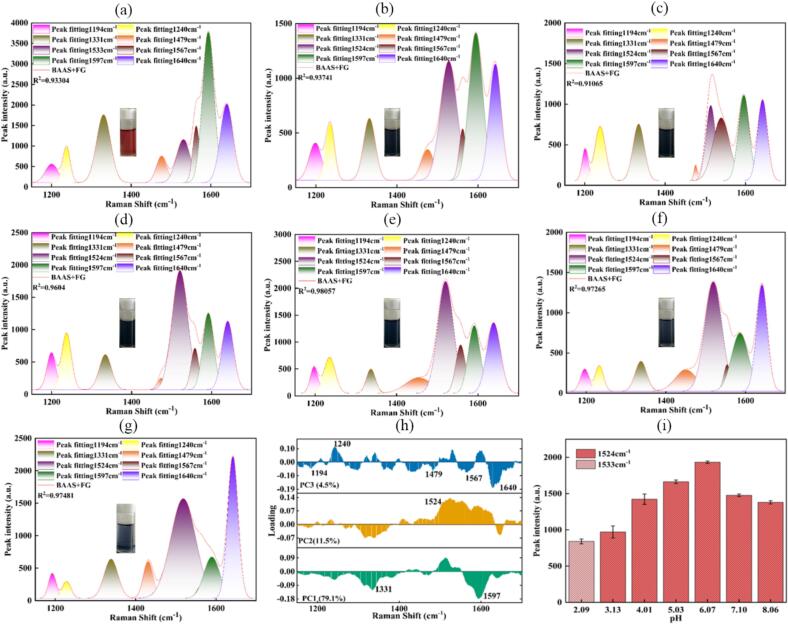
Gaussian multi-peak fitting of spectra (1100–1700 cm^−1^) for BAAS+FG at pH values of 2.09, 3.13, 4.01, 5.03, 6.07, 7.10, and 8.06 (a–g), PCA loading of BAAS+FG Raman spectra at different pH values (h), and intensity variation of characteristic peak at 1524 cm^−1^ across different pH values (i). Note that when pH ≤ 3.13, the 1533 cm^−1^ peak was used for fitting; when pH ≥ 3.72, the 1524 cm^−1^ peak was used for fitting.

For the BAAS+FG system, the Gaussian multi-peak fitting yielded high goodness-of-fit values (R^2^ = 0.91–0.98) across the entire pH range (Fig. 4(a–g)). The fitting approach was tailored to pH conditions: under strong acidic conditions (pH ≤ 2.09), the characteristic peak at 1533 cm^−1^—associated with the flavylium cation—was used, consistent with a red-colored solution. Under weakly acidic to slightly alkaline conditions (pH 3.13–8.06), the characteristic chelate peak at 1524 cm^−1^, corresponding to the blue-black quinonoidal base chelate, was applied. The PCA loading plot (Fig. 4(h)) identified the 1524/1533 cm^−1^ peak as the dominant contributor to the first principal component, highlighting its critical role in responding to pH variation. The intensity of the 1524 cm^−1^ peak increased with rising pH, indicating enhanced chelate formation (Fig. 4(i)). However, under alkaline conditions (pH ≥ 7.10), the basic environment triggered ring-opening degradation of anthocyanins (Duan et al., 2024), disrupting the chelation equilibrium. This resulted in dissociation of complex, accompanied by a lighter solution color, increased turbidity, and a corresponding decrease in the intensity of the 1524 cm^−1^ peak.

**Fig. 5:**
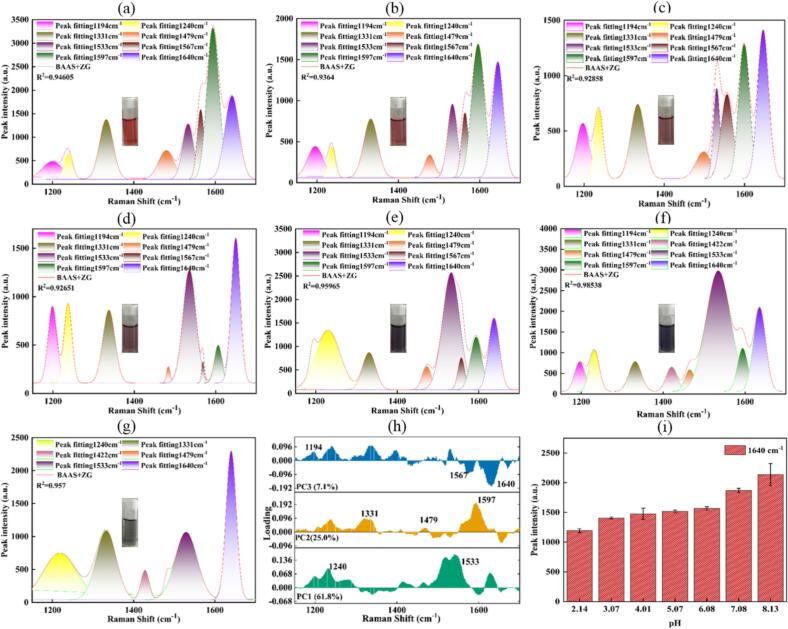
Gaussian multi-peak fitting of spectra (1100–1700 cm^−1^) for BAAS+ZG at pH values of 2.14, 3.07, 4.01, 5.07, 6.08, 7.08, and 8.13 (a–g), PCA loadings of BAAS+FG Raman spectra at different pH values (h), and intensity variation of characteristic peak at 1640 cm^−1^ across different pH values (i).

A related but distinct behavior was observed for the BAAS+ZG system (Fig. 5(a–g)), which also exhibited high fitting accuracies (R^2^ = 0.93–0.99). Across the tested pH range, all characteristic Raman shifts remained consistent with those of BAAS. Visually, the solution color transitioned from purple-red at lower pH to blue-purple above pH 6. Under weakly alkaline conditions (pH = 8.13), anthocyanin degradation and the formation of zinc hydroxide precipitate led to color fading and increased turbidity. The spectral response of the Zn^2+^ system to pH variation resembled that of the Fe^2+^ system. As shown in the PCA loadings (Fig. 5(h)), multiple characteristic peaks—1194, 1240, 1331, 1479, 1524 (1533), 1567, 1597, and notably 1640 cm^−1^—were strongly weighted and exhibited high correlation with pH. A key spectral response was the systematic increase in intensity of the *ν*(C=O) peak at 1640 cm^−1^ with increasing pH (Fig. 5(i)). This enhancement supports the role of Zn^2+^ in stabilizing the quinonoidal base structure, the concentration of which rises with pH. Notably, the Raman peak at 1640 cm^−1^ maintained considerable intensity even under high-pH conditions where color fading occurred due to degradation. This suggests that the Zn^2+^ complex may enhance the Raman scattering intensity of residual quinoidal bases in solution. An alternative explanation is that undegraded chelates retain a highly conjugated and rigid structure, thereby sustaining a strong Raman signal.

### 3.4 Enhanced thermal and UV stability by CG-stabilized BA-Fe^2+^(Zn^2+^)

#### 3.4.1 Zeta potential analysis

**Fig. 6:**
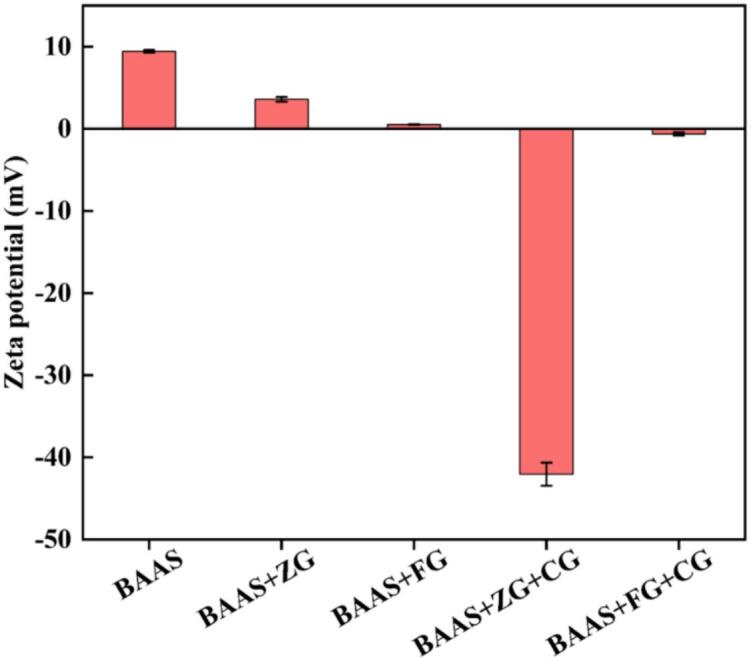
Zeta potential of BAAS, BAAS+ZG(FG) and BAAS+ZG(FG) + CG.

As illustrated in Fig. 6, notable differences were observed in the zeta potentials of BAAS, BAAS+ZG/FG, and BAAS+ZG/FG + CG. Under acidic conditions, pure BAAS exhibited a positive zeta potential of +9.42 ± 0.16 mV, which is mainly attributed to the prevalence of anthocyanins in the flavylium cation form (Fu et al., 2023; Szewczuk et al., 2022). Upon chelation with Zn^2+^ or Fe^2+^, the zeta potential decreased substantially. The value for BAAS+ZG dropped to approximately +3.59 ± 0.28 mV, while that for BAAS+FG approached electrical neutrality (+1.46 ± 0.29 mV). This reduction can be explained by two contributing mechanisms: first, the chelation of metal ions with the ortho-dihydroxyl groups on the anthocyanin B-ring displaces H^+^ ions, resulting in deprotonation and a corresponding loss of positive charge; second, the introduced metal cations partially neutralize the positive charges carried by the flavylium cations. Owing to its stronger chelating capacity—reflected in more coordination sites and higher binding constants—Fe^2+^ was more effective than Zn^2+^ in shielding the positive surface charges of anthocyanins. As a result, the BAAS+FG system approached charge neutrality, indicating poor colloidal stability.

After the introduction of the anionic polysaccharide CG, the two systems displayed distinct zeta potential behaviors. The zeta potential of BAAS+ZG + CG decreased sharply to approximately −42 ± 1.40 mV. This suggests that Zn^2+^ likely served as a bridging element between anthocyanins and CG, while the abundant negatively charged sulfate groups on the CG chains covered the particle surface, inducing strong electrostatic repulsion and thereby significantly improving colloidal stability. In contrast, the zeta potential of BAAS+FG + CG shifted only slightly to a negative value (−1.88 ± 0.20 mV). We hypothesize that Fe^2+^ in the system may be oxidized to Fe^3+^. As a trivalent cation, Fe^3+^ can crosslink multiple anionic sites on both CG and anthocyanins, forming a dense three-dimensional network that effectively encapsulates the anthocyanins. Under this mechanism, system stability is maintained primarily through steric hindrance and structural encapsulation rather than electrostatic repulsion. Hence, despite the low absolute zeta potential, anthocyanin stability was still enhanced.

#### 3.4.2 Degradation kinetic model

The temporal changes in the Raman peak intensity ratio (*I*_1567_/*I*_631_) for BAAS and CG-stabilized BAAS+FG(ZG) systems under thermal and UV irradiation treatments were fitted using zero-, first-, and second-order kinetic models. Corresponding kinetic equations and rate constants were calculated (Table 1), with the best-fit model selected according to the highest coefficient of determination.

**Table 1:** Degradation kinetic equations and *k* values under heat treatment and UV irradiation.

Sample	Heat treatment		UV irradiation	
	*k* (min^−1^)	Fitting Equation	*k* (h^−1^)	Fitting Equation
BAAS	0.0010	1/*C*_t_–1/*C*_0_ = 0.001*x* + 0.0157	0.0097	*C*_0_–*C*_t_ = 0.0097*x*-0.2669
BAAS+FG + CG	0.0002	1/*C*_t_–1/*C*_0_ = 0.0002*x* + 0.0012	0.0039	*C*_0_–*C*_t_ = 0.0039*x*-0.0484
BAAS+ZG + CG	0.0001	1/*C*_t_–1/*C*_0_ = 0.0001*x* + 0.00005	0.0014	*C*_0_–*C*_t_ = 0.0014*x*-0.0305

As shown in Table 1, the thermal degradation of both BAAS and BAAS+FG(ZG) + CG followed second-order kinetics, consistent with previous reports (Zhang et al., 2020).The rate constant (*k*) for BAAS was 0.001 min^−1^, while those for the CG-stabilized BAAS+FG + CG and BAAS+ZG + CG systems were 0.0002 min^−1^ and 0.0001 min^−1^, respectively—representing only one-fifth and one-tenth of the value for BAAS alone. Lower *k* value correspond to slower thermal degradation, indicating that CG effectively decelerates the degradation of BA. The observed *k* values for the CG-stabilized chelates are lower than the reported value of 0.0027 ± 1.31 × 10^−4^ min^−1^ for silkworm pupae protein-encapsulated cyanidin-3-glucoside (C3G) at 80 °C (Attaribo et al., 2020), yet higher than that of the mulberry anthocyanin-ferulic acid complex with a *k* value of 0.00082 min^−1^ (Chen et al., 2022). The stabilization effect is attributed to the strong electronegativity of CG, which facilitates electrostatic interactions with positively charged chelates, resulting in a more stable composite structure. In particular, the cross-linked gel network formed by CG restricts the molecular mobility of BA and reduces its thermal exposure.

In contrast to thermal degradation, UV-induced degradation of BAAS and its CG-stabilized chelates followed zero-order kinetics (Table 1). The *k* values for BAAS+FG + CG and BAAS+ZG + CG under UV irradiation ranged from 0.0014 to 0.0039 h^−1^, significantly lower than that of the BAAS (0.0097 h^−1^). This behavior can be explained by the formation of stable complexes between anthocyanin molecules and polysaccharides—such as cyclodextrins, dextrans, and pectin—via non-covalent interactions, primarily hydrogen bonding and electrostatic attractions. Such intermolecular associations are known to contribute to structural stabilization, particularly between anthocyanins and non-starchy polysaccharides (Zang et al., 2022).

In summary, CG effectively enhances the stability of metal-chelated anthocyanins, retarding degradation under both thermal and UV stress. These findings underscore the potential of Raman spectroscopy as an efficient, non-destructive tool for monitoring the stability of multicomponent nutrient systems in food.

#### 3.4.3 Degradation rate analysis

Raman spectra were collected at predetermined intervals. Following Gaussian multi-peak fitting, the degradation rate of BA in BAAS, BAAS+FG + CG, and BAAS+ZG + CG under thermal and UV treatments was calculated using Formula (1). Multiple fitting models were evaluated, and the one with the highest R^2^ value was chosen as the degradation rate equation.

**Fig. 7:**
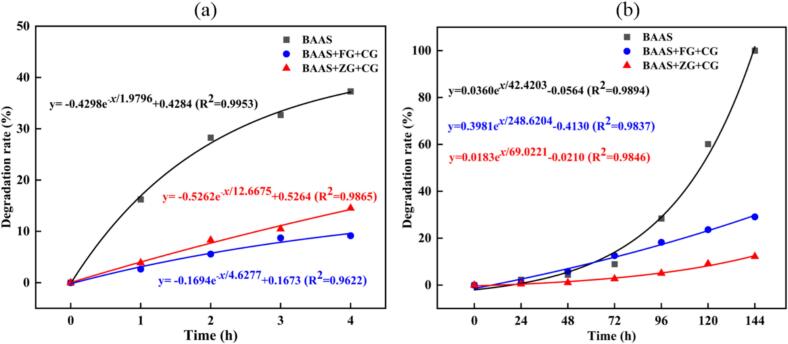
Degradation rate of heat treatment (a) and UV irradiation (b) on CG-stabilized BA-Fe^2+^(Zn^2+^) chelates.

Fig. 7(a) presents the degradation behavior of BA in different systems during continuous heating at 80 °C. The degradation rate of BAAS alone was best described by an exponential model (R^2^ = 0.9953), marked by an initial rapid phase followed by a slower decay. In contrast, anthocyanin degradation in the CG-stabilized systems followed a more linear trend over time, with R^2^ values of 0.9622 for BAAS+FG + CG and 0.9865 for BAAS+ZG + CG. Heat treatment readily induces anthocyanin degradation, causing cleavage of the benzopyrylium group to form chalcone, which further degrades into simple aromatic compounds containing hydroxyl, aldehyde, methoxy, or carboxyl functional groups (Duan et al., 2024). At each measured time point, the degradation rates of CG-stabilized BAAS+FG + CG and BAAS+ZG + CG were significantly lower than that of the control BAAS. For example, after 240 min of heating at 80 °C, the degradation rate of BAAS reached nearly 40%, whereas values of BAAS+FG + CG and BAAS+ZG + CG were only 9% and 14%, respectively.

Under continuous U*V* irradiation, the degradation patterns of BA diverged considerably from those under thermal treatment (Fig. 7(b)). The initial degradation rate was relatively low but increased markedly in later phase, particularly in the BAAS system. The UV-induced degradation profiles were well fitted, with R^2^ values of 0.9894 for BAAS, 0.9837 for BAAS+FG + CG, and 0.9846 for BAAS+ZG + CG. Increased light intensity and prolonged exposure time are known to accelerateanthocyanin photodegradation (Rakić & Poklar Ulrih, 2021), with UV radiation particularly inducing oxidation of phenolic hydroxyl groups. Again, the degradation rates in CG-stabilized systems remained significantly lower than that of the control throughout the irradiation period. After 144 h of exposure, BAAS was nearly fully degraded, whereas degradation rates for BAAS+FG + CG and BAAS+ZG + CG reached only 29% and 12%, respectively.

These results further demonstrate that anthocyanin-metal chelates can interact with certain polysaccharides to enhance the color stability of the complexes (Huang et al., 2023). Accordingly, CG effectively improves the photothermal stability of BA-FG and BA-ZG chelates, highlighting its strong potential for use in nutrient-fortified food systems.

## 4 Conclusions

This study demonstrates that BA form reversible, pH-dependent chelates with Fe^2+^ and Zn^2+^, a process effectively monitored using Raman spectroscopy. The characteristic Raman bands at 1524 cm^−1^ (for Fe^2+^) and 1640 cm^−1^ (for Zn^2+^) provide reliable markers for tracking chelate formation and dissociation. The addition of CG markedly improved the stability of these complexes under both thermal and UV stress, reducing degradation rate constants by up to 90% under certain conditions. This stabilization is attributed to the formation of a protective polysaccharide network through electrostatic and other non-covalent interactions, which limits molecular mobility and mitigates degradation. These findings highlight the potential of anthocyanin–metal–polysaccharide ternary complexes as stable colourant and nutrient delivery systems in functional foods. They also confirm Raman spectroscopy as a powerful analytical tool for quality control and stability assessment in complex food matrices. Future studies should investigate a broader range of metal ions—such as Cu^2+^, Mg^2+^, Ca^2+^—to further elucidate the diversity of anthocyanin–metal interactions. It is also essential to evaluate these complexes in real food matrices, such as beverages and baked goods, under common storage conditions to assess their practical applicability.

## CRediT authorship contribution statement

**Yixiao Wang:** Writing – original draft, Visualization, Validation, Formal analysis, Data curation. **Menglong Ma:** Investigation, Formal analysis, Data curation. **Haitao Fu:** Investigation, Data curation. **Xianhao Ding:** Formal analysis, Data curation. **Chuchu Duan:** Investigation. **Xiaodan Liu:** Validation, Investigation, Conceptualization. **Huang Dai:** Validation, Investigation. **Ning Yang:** Visualization, Data curation. **Fuwei Pi:** Supervision, Resources. **Jiahua Wang:** Writing – review & editing, Supervision, Project administration, Methodology, Funding acquisition. **Ming Zhang:** Writing – review & editing, Resources, Funding acquisition.

## Declaration of competing interest

The authors declare that they have no known competing financial interests or personal relationships that could have appeared to influence the work reported in this paper.

## Data Availability

Data will be made available on request.
